# Pro-inflammatory role of NLRP3 inflammasome in experimental sterile corneal inflammation

**DOI:** 10.1038/s41598-019-46116-9

**Published:** 2019-07-03

**Authors:** Hiroaki Shimizu, Tohru Sakimoto, Satoru Yamagami

**Affiliations:** Department of Visual Sciences, Division of Ophthalmology, Nihon University School of Medicine, Tokyo, Japan

**Keywords:** Molecular medicine, Inflammatory diseases

## Abstract

We evaluated the role of NLR family pyrin domain containing 3 (NLRP3) inflammasome in sterile corneal inflammation caused by lipopolysaccharide (LPS) or alkali burns in C57BL6 mice or NLRP3 KO (*Nlrp3*^*−/−*^) mice. Various molecules related to the NLRP3 inflammasome were upregulated in C57BL6 mice after both alkali burn injury and LPS treatment. After alkali burn injury, the corneal opacity grade was significantly reduced in *Nlrp3*^*−/−*^ mice compared with C57BL6 mice. In *Nlrp3*^*−/−*^ mice, Gr-1 immunoreactivity and MMP-9 mRNA expression in the corneal stroma were significantly reduced by both LPS treatment and alkali burn injury. Quantitative PCR and immunohistochemistry revealed that IL-1β and MMP-9 expression in the corneal stroma were down-regulated in *Nlrp3*^*−/−*^ mice with both alkali burn injury and LPS treatment. These findings suggest that the NLRP3 inflammasome has a pro-inflammatory effect in the cornea by recruiting neutrophils to sites of inflammation.

## Introduction

There is accumulating evidence indicating that inflammation in the absence of pathogens, i.e., sterile inflammation, is mediated by the inflammasome, a large multiprotein complex in the cytosol that regulates production of the pro-inflammatory cytokine interleukin (IL)-1β production^1^. The protein complex contains members of the nucleotide-binding oligomerization domain-like receptor (NLR) family, as well as the PYD and CARD domain containing adaptor protein (PYCARD, ASC) that recruits inflammatory pro-caspase-1 (also known as IL-1β-converting enzyme). After sensing of appropriate ligands and activation of the inflammasome, cleavage and activation of caspase-1 occurs, resulting in the processing of IL-1β and IL-18 to biologically active forms that are secreted. To date, the most extensively studied and characterized inflammasome is that containing NLR family pyrin domain containing 3 (NLRP3). A wide variety of stimuli can activate NLRP3, including pathogen-associated molecular patterns (PAMPs) such as lipopolysaccharide, peptidoglycan, and double-stranded DNA/RNA. NLRP3 is also activated by danger-associated molecular patterns (DAMPs) associated with cellular stress, including extracellular ATP, asbestos, amyloid-β, and extracellular DNA or RNA, which can initiate and sustain a sterile inflammatory response. Importantly, most DAMPs are released extracellularly after tissue injury. When recognized at the cell surface, these molecules activate toll-like receptors, while intracellular recognition leads to inflammasome activation via NLR family members^2,3^_._

Inflammation of the ocular surface and/or non-infectious corneal ulcers, such as Mooren’s ulcer, corneal ulcers associated with collagen vascular diseases, sequelae of eye injury including chemical burns, and secondary ulcers caused by various ocular surface diseases, can lead to a variety of sightthreatening complications. Despite the numerous conditions that can cause sterile corneal ulcers, effective and safe anti-inflammatory therapy remains one of the unmet medical needs for ocular surface inflammation^4^.

This study was performed to investigate the role of NLRP3 in sterile corneal inflammation. We employed alkali burn injury and application of lipopolysaccharide (LPS) to induce sterile inflammation of the cornea in wild-type mice and NLRP3 KO (knockout, *Nlrp3*^*−/−*^) mice, and investigated NLRP3-dependent corneal opacity, neutrophil infiltration, and up-regulation of proinflammatory molecules. Our results may prove useful for the future development of topical low molecular weight drugs to treat corneal inflammation.

## Results

### Up-regulation of various inflammasome-related molecules, including NLRP3, after corneal alkali burn injury

Corneal alkali burn injury not only causes corneal neovascularization and massive infiltration of inflammatory cells, but also results in tissue destruction with ulceration/perforation and massive apoptosis in the corneal stroma^5^. Therefore, we used corneal alkali burn injury in mice as a suitable model for studying the impact of various DAMPs on expression of inflammasome-related molecules in the cornea.

**Figure 1:**
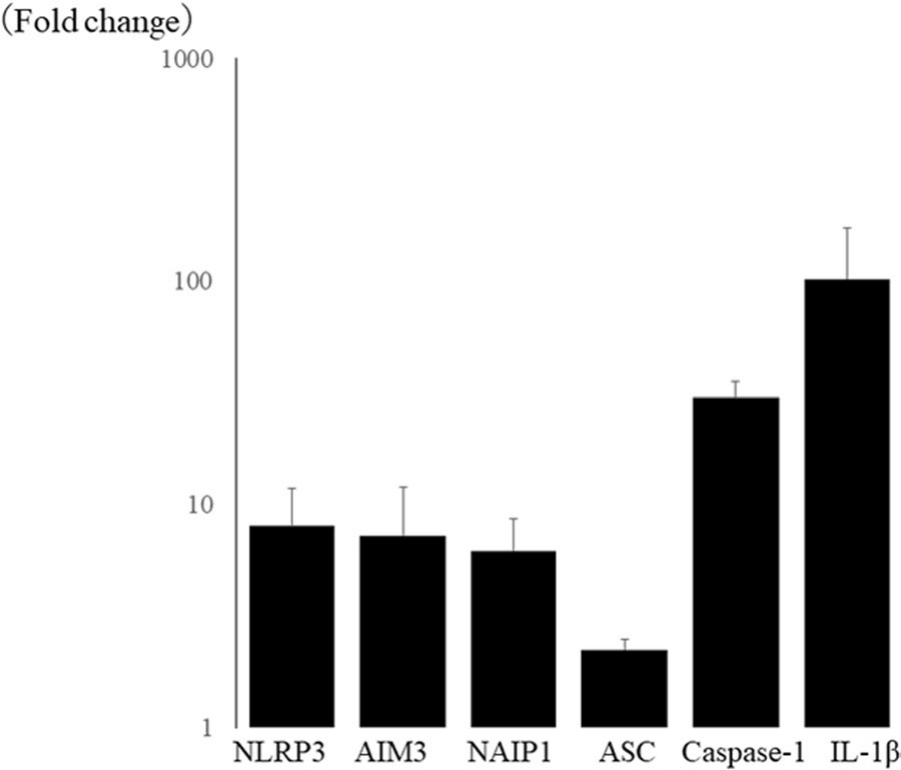
Inflammasome-related molecules are up-regulated in corneal alkali burn using PCR array. Among NLR family, NLRP3 (8.0 ± 3.9 fold) is most up-regulated, and AIM(absent in melanoma)3 (7.2 ± 4.8 fold) and NAIP1 (neuronal apoptosis inhibitory protein 1) (6.2 ± 2.5 fold) are also up-regulated. Other inflammasome components, IL-1β (102.4 ± 71.1 fold), caspase-1 (30.1 ± 5.4 fold), and apoptosis-associated speck-like protein containing a carboxy-terminal CARD (ASC) (2.2 ± 0.3 fold) were up-regulated in alkali burned cornea.

Using a PCR array, we found that various inflammasome-related genes were up-regulated in the cornea after alkali burn injury compared with the intact cornea. Among NLR family members, NLRP3 was most strongly up-regulated (8.0 ± 3.9 fold). Other NLR family members, such as absent in melanoma (AIM)3 (7.2 ± 4.8 fold) and neuronal apoptosis inhibitory protein (NAIP)1 (6.2 ± 2.5 fold) were also up-regulated, as were other components of the inflammasome, including IL-1β (102.4 ± 71.1 fold), caspase-1 (30.1 ± 5.4 fold), and apoptosis-associated speck-like protein containing a carboxy-terminal CARD (ASC) (2.2 ± 0.3 fold) (Fig. 1). These findings suggested that NLRP3 could be the pivotal molecule in experimental corneal inflammation.

### Corneal opacity caused by alkali burn is significantly diminished in NLRP3 KO mice cornea

**Figure 2:**
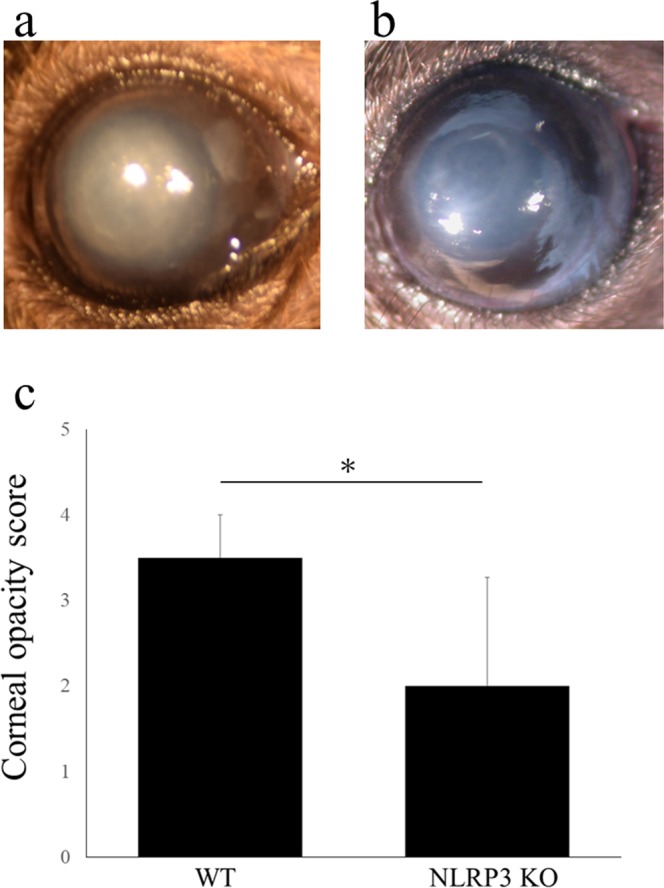
Corneal opacity induced by alkali burn is suppressed in NLRP3 KO mice cornea. Representative photographs are shown. Compared with WT mice (a, opacification grade 4; intense stromal opacity, only outline of pupil is visible), the opacification is suppressed in NLRP3 KO mice (b, opacification grade 2; minimal to moderate opacity including corneal stroma). Using opacification grade, it is shown that corneal opacity in NLRP3 KO mice (**b**) is significantly diminished compared with WT mice (**a**) (N = 10 each, WT mice; 3.5 ± 0.5, NLRP3 KO mice; 2.0 ± 1.2, *P < 0.05, MannWhitney *U*-test) on day 7 after alkali burn injury (**c**).

Next, we investigated NLRP3 KO mice to assess the role of NLRP3 after corneal alkali burn injury. Compared with wild-type (WT) mice (Fig. 2a), the grade of corneal opacity was significantly lower in NLRP3 KO mice (Fig. 2b) on day 7 after alkali burn injury (N = 10 each, 3.5 ± 0.5 vs. 2.0 ± 1.2, respectively, *P < 0.05) (Fig. 2c)

### Reduced corneal neutrophil infiltration in NLRP3 KO mice after alkali burn injury

**Figure 3:**
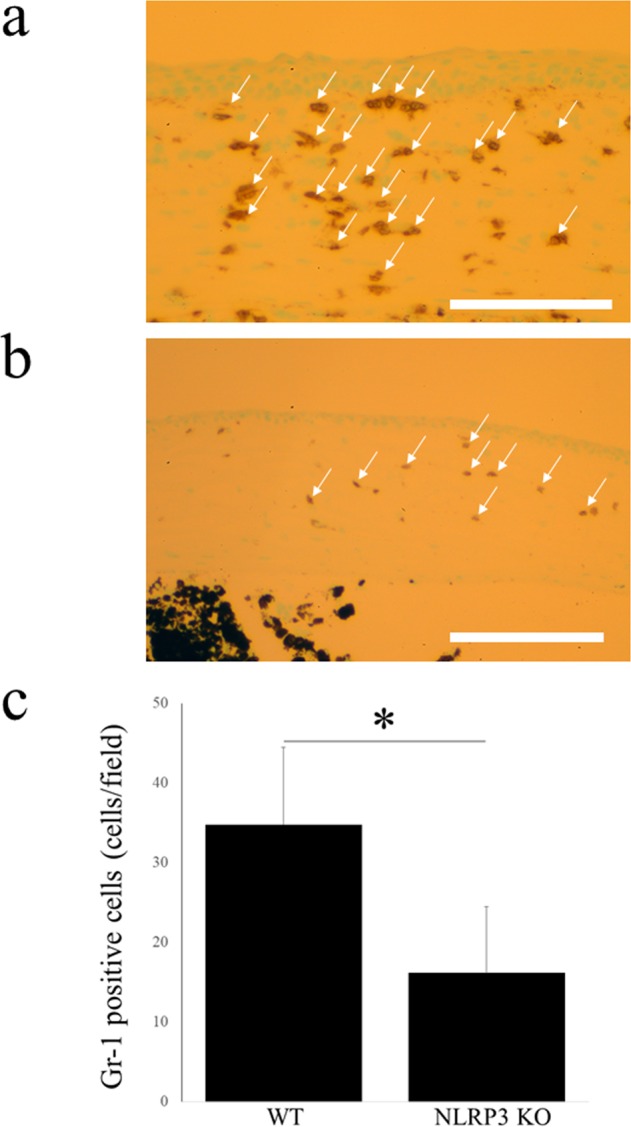
Neutrophil infiltration is diminished after alkali burn injury in NLRP3 KO mice cornea. Compared with WT mice cornea (**a**), Gr-1-positive cells (arrows), which account for neutrophil infiltrations, are significantly fewer in NLRP3 KO mice cornea (**b**) (N = 5 each, WT mice; 34.8 ± 9.7 cells/field, NLRP3 KO mice; 16.1 ± 8.3 cells/field, *P < 0.01, ManneWhitney *U*-test) (**c**). Bar = 40 μm.

At 7 days after alkali burn injury, neutrophils labeled by Gr-1 were extensively observed infiltrating the corneal stroma in WT mice (Fig. 3a). However, neutrophil infiltration of the corneal stroma was significantly diminished in NLRP3 KO mice (Fig. 3b) at the same time after alkali burn injury (N = 5 each, 34.8 ± 9.7 cells/field vs. 16.1 ± 8.3 cells/field, respectively, *P < 0.01) (Fig. 3c).

### Corneal expression of pro-inflammatory chemokine and cytokine genes is suppressed in NLRP3 KO mice after alkali burn injury

**Figure 4:**
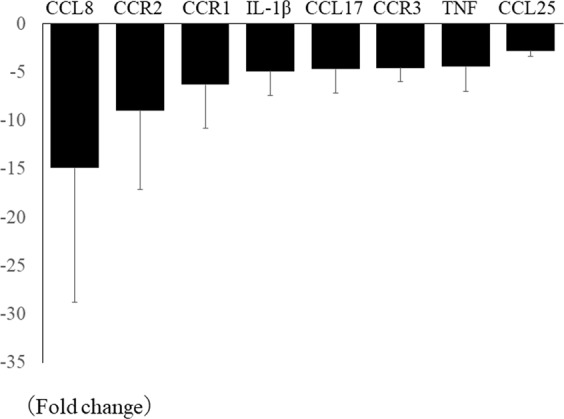
Pro-inflammatory chemokine and cytokine gene expressions are suppressed in NLRP3 KO mice cornea after alkali injury. Several pro-inflammatory chemokine and cytokine gene expressions, CCL8 (−14.9 ± 13.9 fold), CCR2 (−8.9 ± 8.1 fold), CCR1 (−6.3 ± 4.4 fold), IL-1β (−4.9 ± 2.5 fold), CCL17 (−4.7 ± 2.5 fold), CCR3 (−4.6 ± 1.4 fold), TNF (−4.4 ± 2.6 fold), and CCL25 (−2.8 ± 0.5 fold) are down-regulated in NLRP3 KO mice cornea compared with those in WT mice using PCR array.

Compared with WT mice, PCR array analysis revealed down-regulation of the corneal expression of several pro-inflammatory chemokine and cytokine genes in NLRP3 KO mice, including CCL8 (−14.9 ± 13.9 fold), CCR2 (−8.9 ± 8.1 fold), CCR1 (−6.3 ± 4.4 fold), IL-1β (−4.9 ± 2.5 fold), CCL17 (−4.7 ± 2.5 fold), CCR3 (−4.6 ± 1.4 fold), TNF (−4.4 ± 2.6 fold), and CCL25 (−2.8 ± 0.5 fold) (Fig. 4).

### Corneal expression of MMP-9 mRNA is down-regulated in NLRP3 KO mice after alkali burn injury

**Figure 5:**
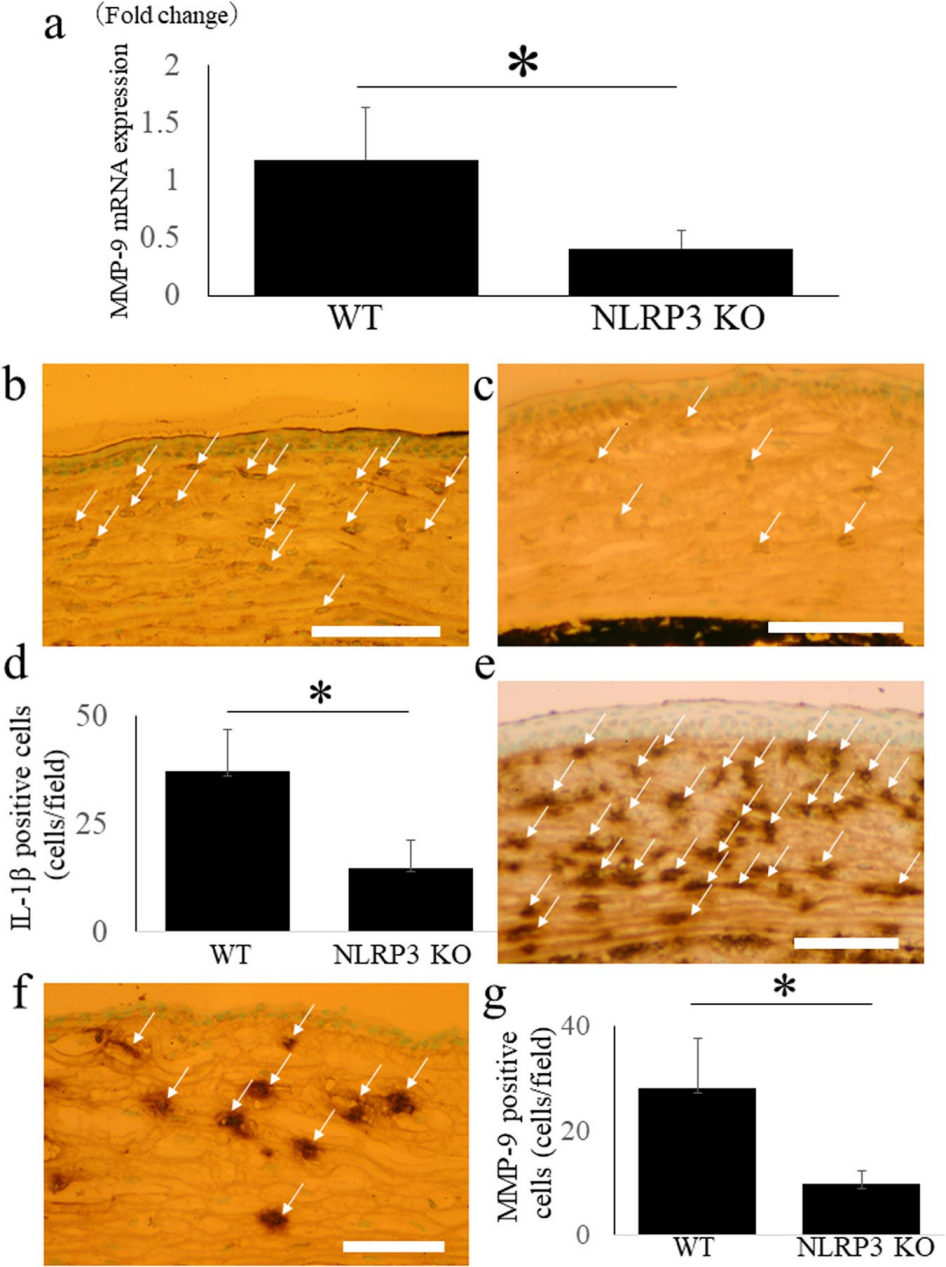
MMP-9 mRNA expression, MMP-9 and IL-1β immunoreactivity are down-regulated in NLRP3 KO mice cornea after alkali injury. MMP-9 mRNA expression is significantly down-regulated in NLRP3 KO mice cornea compared with those in WT mice after corneal alkali burn using quantitative RT-PCR (N = 8 each, WT mice; 1.2 ± 0.5 fold, NLRP3 KO mice; 0.4 ± 0.1 fold, P < 0.05). (**a**) In WT mice, extensive immunoreactivities of IL-1β (arrows) are observed in corneal stroma (**b**), but they are diminished in NLRP3 KO mice. (**c**) The cell counts of IL-1β positive cells (**d**) (N = 8 each, WT mice; 28.2 ± 9.6 cells/field, NLRP3 KO mice; 9.8 ± 2.6 cells/field, P < 0.01) has significant difference between WT mice and NLRP3 KO mice. Bar = 40 μm. Compared with WT mice (**e**), MMP-9 immunoreactivity (arrows) is diminished in NLRP3 KO mice. (**f**) The cell counts of MMP-9 positive cells (**g**) (N = 8 each, WT mice; 37.3 ± 9.7 cells/field, NLRP3 KO mice; 14.9 ± 6.3 cells/field, P < 0.01) are significantly decreased in NLRP3 KO mice. Bar = 40 μm.

MMP-9 is a potent cause of corneal neovascularization, ulceration, and inflammation. It is widely accepted that MMP-9 is released by resident corneal cells and infiltrating neutrophils, and that its release is regulated by IL-1β via the NF-κB and AP-1 pathways^6,7^. Therefore, it seems likely that MMP-9 would be an important downstream target of the NLRP3 inflammasome via IL-1β in the cornea. Quantitative RT-PCR revealed that corneal expression of MMP-9 mRNA was significantly down-regulated in NLRP3 KO mice compared with WT mice after alkali burn injury (N = 8 each, 0.4 ± 0.1 fold vs. 1.2 ± 0.5 fold, respectively, *P < 0.05) (Fig. 5a).

### Down-regulation of corneal IL-1β and MMP-9 immunoreactivity in NLRP3 KO mice after alkali burn injury

We next evaluated MMP-9 and IL-1β protein expression by using immunohistochemistry. In WT mice, immunoreactivity for IL-1β (Fig. 5b) and MMP-9 (Fig. 5e) was extensively observed in the corneal stroma, but such immunoreactivity was diminished in NLRP3 KO mice (Fig. 5c,f, respectively). There was a significant difference between WT mice and NLRP3 KO mice with respect to the number of IL-1β-positive cells in the corneal stroma (Fig. 5d) (N = 8 each, WT mice; 28.2 ± 9.6 cells/field, NLRP3 KO mice; 9.8 ± 2.6 cells/field, *P < 0.01), as well as the number of MMP-9 positive cells (Fig. 5g) (N = 8 each, WT mice; 37.3 ± 9.7 cells/field, NLRP3 KO mice; 14.9 ± 6.3 cells/field, *P < 0.01).

### Application of LPS after corneal epithelial ablation induces up-regulation of NLRP3 inflammasome components

**Figure 6:**
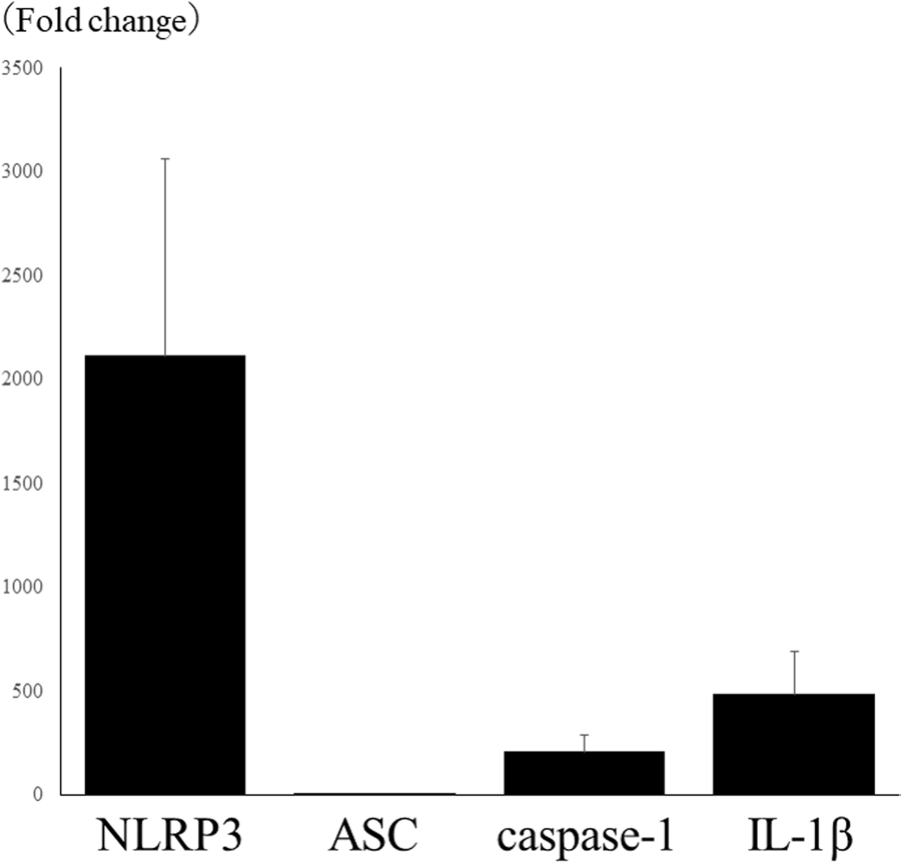
LPS application after corneal epithelial ablation induces up-regulation of NLRP3 inflammasome components. NLRP3 (2113.1 ± 950.4 fold), IL-1β (485.9 ± 208.7 fold), and caspase-1 (210.5 ± 79.2 fold) are up-regulated in the cornea of 2 days after instillation of LPS compared with those of non-treated cornea using quantitative RT-PCR. ASC (1.3 ± 0.3 fold) is not up-regulated in LPS applied cornea.

LPS is a representative PAMP protein, and its application after corneal epithelial ablation induces infiltration of leukocytes and corneal inflammation^8,9^. Using quantitative RT-PCR, we found upregulation of the expression of NLRP3 (2113.1 ± 950.4 fold), IL-1β (485.9 ± 208.7 fold), and caspase-1 (210.5 ± 79.2 fold) in the corneal stroma at 2 days after LPS treatment compared with untreated corneas (Fig. 6). In contrast, there was no up-regulation of ASC expression (1.3 ± 0.3 fold) after LPS treatment.

### NLRP3 KO mice show reduced neutrophil infiltration after LPS treatment

**Figure 7:**
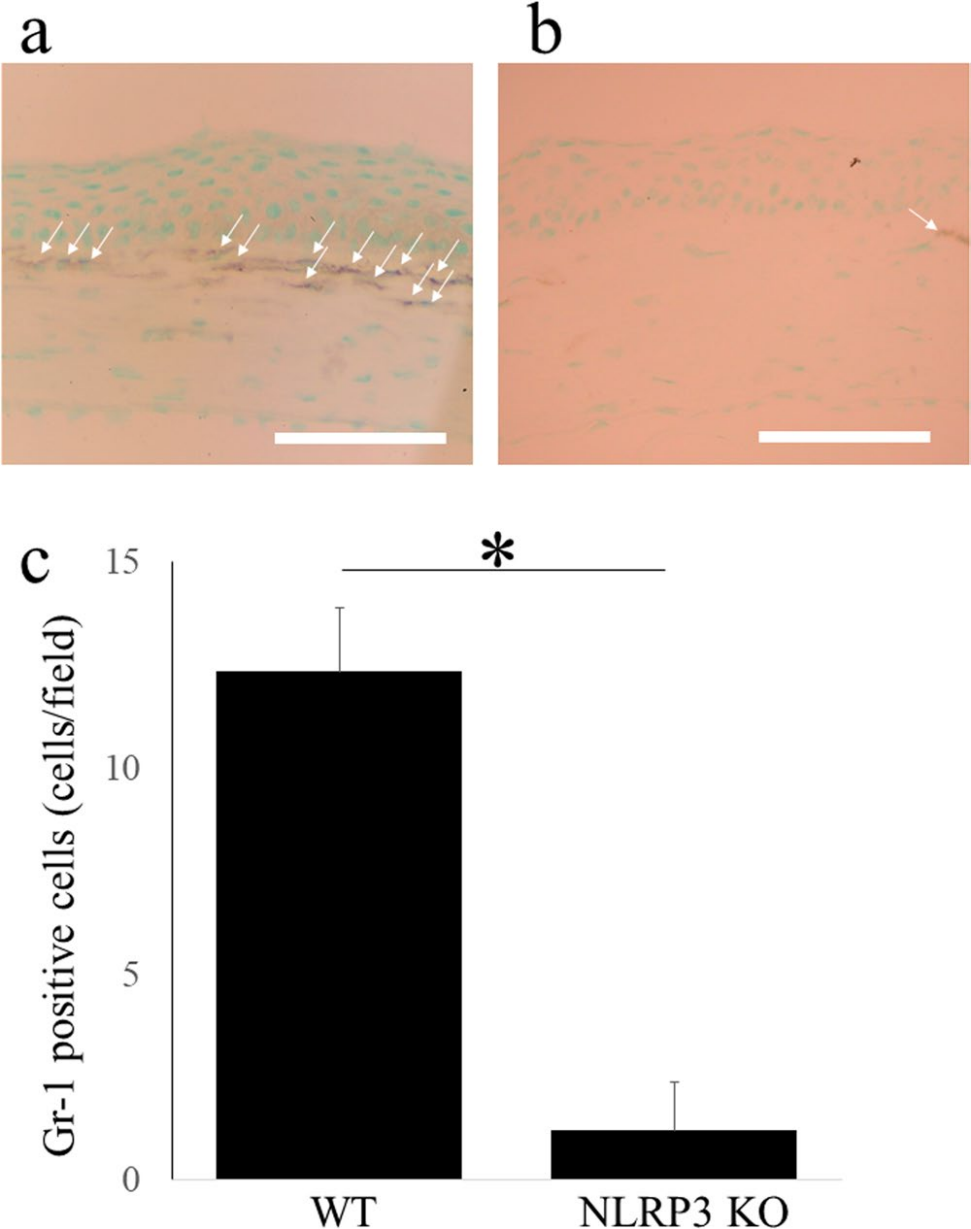
Neutrophil accumulation after LPS application is diminished in NLRP3 KO mice. Gr-1-positive cells (arrows), which account for neutrophil, are infiltrated in anterior stroma of WT mice 2 days after LPS application. (**a**) In NLRP3 KO mice (**b**), this accumulation is canceled, and the Gr-1-positive cells are significantly fewer compared with those of WT mice (N = 3 each, WT mice; 12.3 ± 1.5 cells/field, NLRP3 KO mice; 1.2 ± 1.1 cells/field, P < 0.01, ManneWhitney *U*-test) (**c**). Bar = 40 μm.

Two days after LPS treatment, infiltration of Gr-1-positive cells (corresponding to neutrophils) was seen in the anterior corneal stroma of WT mice (Fig. 7a). In contrast, infiltration of neutrophils was suppressed in NLRP3 KO mice and significantly fewer Gr-1-positive cells were seen than in WT mice (N = 3 each, 1.2 ± 1.1 cells/field vs. 12.3 ± 1.5 cells/field, respectively, *P < 0.01) (Fig. 7b,c).

### Down-regulation of MMP-9 mRNA expression in NLRP3 KO mice after LPS treatment

**Figure 8:**
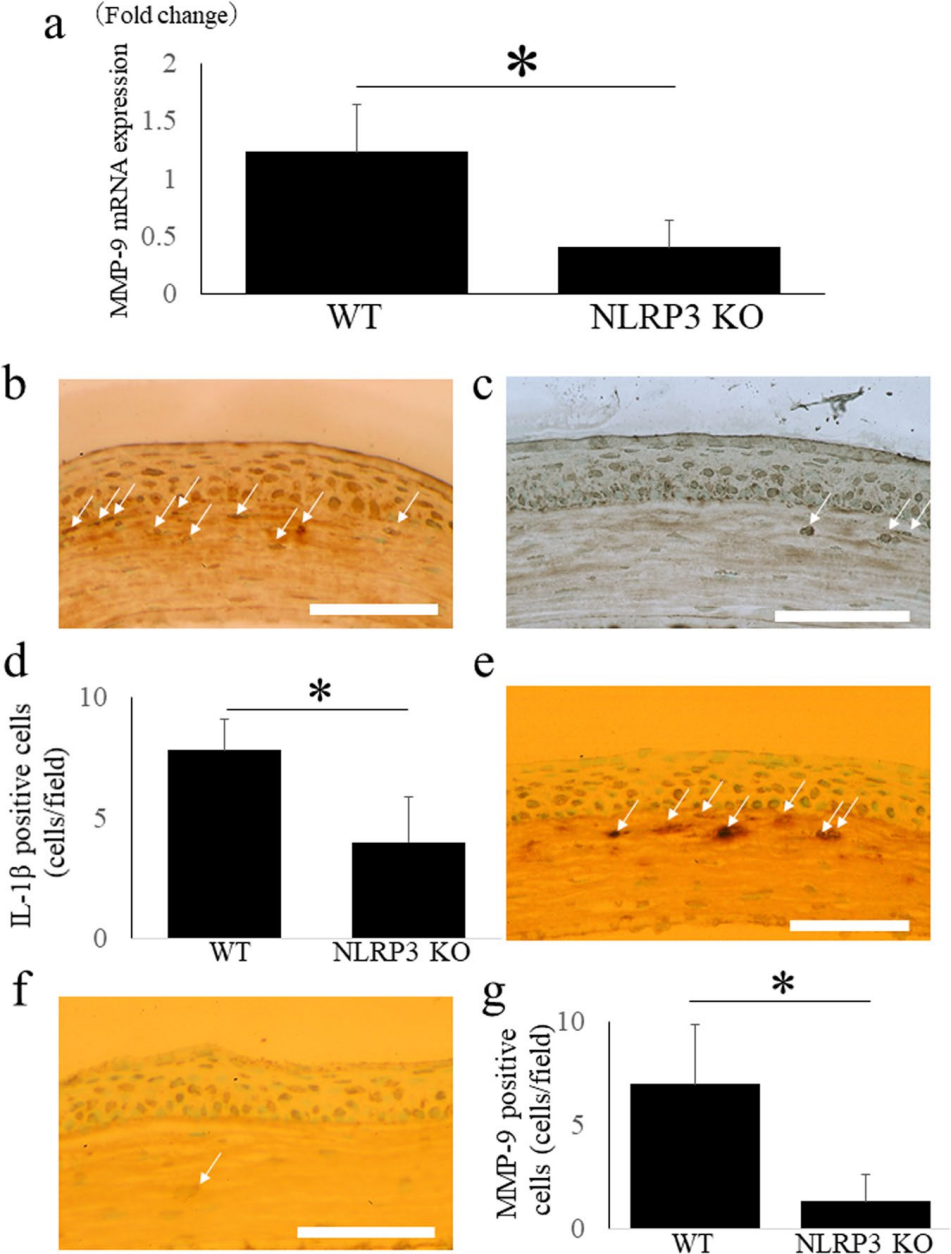
MMP-9 mRNA expression, MMP-9 and IL-1β immunoreactivity are down-regulated in NLRP3 KO mice cornea after alkali injury. MMP-9 was significantly down-regulated in NLRP3 KO mice cornea compared with those in WT mice 2 days after LPS application using a quantitative RT-PCR (WT mice; 1.2 ± 0.4 fold, NLRP3 KO mice; 0.4 ± 0.3 fold, P < 0.05). (**a**) In WT mice, immunoreactivity of IL-1β (**b**) is observed in corneal anterior stroma, but it is almost absent in NLRP3 KO mice (**c**) (arrows). The cell counts of IL-1β positive cells (**d**) (N = 8 each, WT mice; 28.2 ± 9.6 cells/field, NLRP3 KO mice; 9.8 ± 2.6 cells/field, P < 0.01) indicates the significant difference between WT mice and NLRP3 KO mice. Bar = 40 μM. Compared with WT mice (**e**), MMP-9 positive cells (arrows) observed in corneal anterior stroma are diminished in NLRP3 KO mice. (**f**) The cell counts of MMP-9 positive cells (**g**) (N = 8 each, WT mice; 37.3 ± 9.7 cells/field, NLRP3 KO mice; 14.9 ± 6.3 cells/field, P < 0.01) are significantly decreased in NLRP3 KO mice. Bar = 40 μm.

Quantitative RT-PCR demonstrated that expression of MMP-9 was significantly down-regulated in the corneas of NLRP3 KO mice compared with WT mice at 2 days after LPS treatment (N = 3 each, 0.4 ± 0.3 fold vs. 1.2 ± 0.4 fold, respectively, *P < 0.05) (Fig. 8a).

### Down-regulation of IL-1β and MMP-9 immunoreactivity in NLRP3 KO mice after LPS treatment

Finally, we evaluated immunoreactivity for IL-1β and MMP-9 after LPS treatment. In WT mice, immunoreactivity for both IL-1β (Fig. 8b) and MMP-9 (Fig. 8e) was observed in the anterior corneal stroma, but it was almost absent in NLRP3 KO mice (Fig. 8c,f, respectively). The IL-1β-positive cell count (Fig. 8d) (N = 8 each, WT mice; 28.2 ± 9.6 cells/field, NLRP3 KO mice; 9.8 ± 2.6 cells/field, P < 0.01) and the MMP-9-positive cell count (Fig. 8g) (N = 8 each, WT mice; 37.3 ± 9.7 cells/field, NLRP3 KO mice; 14.9 ± 6.3 cells/field, P < 0.01) both showed a significant difference between the 2 strains of mice.

## Discussion

This study detected NLRP3-dependent neutrophil infiltration of the cornea in 2 mouse models of sterile corneal inflammation. In the corneal alkali burn injury model, various NLRP3 inflammasome-related molecules were up-regulated, and NLRP3 knockout led to significant reduction of corneal opacity, neutrophil infiltration, and expression of pro-inflammatory molecules including MMP-9. Similar up-regulation of NLRP3 inflammasome-related molecules was seen in the LPS model, while NLRP3 KO mice showed significant reduction of neutrophil infiltration and expression of pro-inflammatory molecules including MMP-9. Based on these findings, we conclude that the NLRP3 inflammasome is essential for development of sterile corneal inflammation.

Activation of the NLRP3 inflammasome and IL-1β processing have been studied extensively in monocytes, macrophages, and dendritic cells^10,11^. However, although neutrophils are the most prominent infiltrating cells in the early stage of infection or inflammation, the contribution of neutrophils to inflammasome activation and IL-1β production is less well understood. Neutrophils are a source of pro-inflammatory cytokines and other inflammatory molecules, including IL-1β, IFN-γ, IL-17, and MMP-9. Neutrophils are rapidly recruited to the site of inflammation and are generally considered to be short-lived leukocytes that die by apoptosis in the circulation. However, there have been some reports that neutrophils can survive longer at sites of inflammation where they are stimulated by pro-inflammatory cytokines^12^. IL-1β is a cytokine with major roles in inflammation and innate immune response. In general, it is produced by activated monocytes, macrophages, and dendritic cells, after which it induces the production of chemokines or cytokines such as TNFα and IL-6, or proteases such as MMPs, in association with recruitment of neutrophils and proliferation of resident cells (mainly fibroblasts). In our present results, we found that various cytokines and chemokines were suppressed in NLRP3 KO mice with alkali burn injury (Fig. 4). Among these, IL-1β is the major inflammasome-related molecule, while the chemokine receptors (CCR1, CCR2, and CCR3) are involved in chemotaxis of white blood cells, including neutrophils^13,14^. Although the biological role, mechanism of expression, and cellular source of CCL8 under physiological or pathological conditions are poorly understood, recent reports have suggested that it may have potent pro-inflammatory activity^15,16^. Therefore, it seems reasonable to conclude that suppression of corneal neutrophil infiltration/inflammation in NLRP3 KO mice after alkali burn injury was related to down-regulation of these chemokines/chemokine receptors.

It was reported that circulating mitochondrial DAMPs can directly activate neutrophils to induce neutrophil-mediated organ injury and sepsis^17,18^. A pro-inflammatory role of neutrophils in inflammation has also been identified in NLRP3 KO mice^19–21^, but the behavior of neutrophils differs between NLRP3 KO models of infection and inflammation. Nlrp3^*−/−*^ neutrophils show decreased autophagy and augmented phagocytosis leading to survival in polymicrobial sepsis, indicating pleiotropic effects of neutrophil regulation under NLRP3 inflammasome signaling^22^. Neutrophils have been shown to induce degradation of collagen in the corneal stromal by activating corneal fibroblasts and inducing MMP secretion by these fibroblasts, with fibroblast activation being dependent on neutrophil-derived IL-1^23^. Infiltrating neutrophils are a major source of MMP-9, which is associated with the extent of corneal inflammation^24^. In corneal epithelial cells, IL-1 acts via the NF-kB, Smad, p38, and JNK signaling pathways to mainly target a specific region of the MMP-9 promoter. Therefore, neutrophils and resident corneal cells are involved in IL-1 secretion, subsequent MMP-9 production, and destruction of the corneal stroma. In our present study, both gene expression and protein expression of IL-1β and MMP-9 were down-regulated in the corneas of NLRP3 KO mice after both alkali burn injury and LPS application.

Indeed, a previous study demonstrated that neutrophils are the key player in corneal pathophysiology, in association with activation of the NLRP3 inflammasome pathway^25^. Extracellular ATP is a DAMP that is an important inducer of inflammasomes, and it promotes a rapid increase of IL-1β secretion by human and murine neutrophils via the P2X7 receptor-NLRP3 inflammasome axis, with neutrophils being the predominant source of IL-1β processed by the NLRP3 inflammasome in a murine model of acute corneal infection by S. pneumoniae^26^. On the contrary, opposite results have been obtained with regard to viral infection of the cornea. Gimenez *et al*. compared the response to ocular infection with herpes simplex type 1 between NLRP3 KO mice and WT mice. Surprisingly, stromal keratitis manifested an earlier onset and was more severe in NLRP3 KO mice than WT mice, along with significantly increased early neutrophil infiltration and corneal neovascularization, while suppression of early neutrophil infiltration prevented the early onset of keratitis and reduced the level of cleaved IL-1β in NLRP3 KO mice^27^. These results indicate that NLRP3 has immunoregulatory and modulatory roles in corneal pathophysiology.

Previous studies have demonstrated that exposure of the abraded corneal surface to LPS induces neutrophil recruitment to the corneal stroma in animal models^13^. Because LPS is well known to induce activation of the NLRP3 inflammasome, mice with LPS treatment of the cornea seem to be a useful model for investigating corneal inflammation induced by neutrophil infiltration and activation of NLRP3-mediated pro-inflammatory processes. Alkali burn injury of the cornea provokes activation of keratocytes and epithelial cells, as well as massive infiltration of inflammatory cells such as neutrophils and macrophages^28^. Both activated resident cells and infiltrating inflammatory cells are involved in alkali burn-mediated corneal damage and can provoke breakdown of the basement membrane by secretion of MMPs, contributing to pathogenic ulceration and stromal perforation^29^. Therefore, we considered the 2 mouse models used in this study to be the most relevant for investigating the role of the NLRP3 inflammasome in sterile corneal inflammation. Although PAMPs and DAMPs are known to induce activation of the inflammasome in various types of sterile inflammation, their role in the cornea is not yet fully understood. Using LPS as a representative PAMP, we clearly showed a pro-inflammatory effect of the NLRP3 inflammasome on neutrophil infiltration. Corneal alkali burn injury causes extensive tissue destruction, including ulceration/perforation and massive apoptosis in the corneal stroma. Therefore, we used a mouse model of corneal alkali burn injury as the optimum method for studying the impact of various DAMPs on inflammasome-related molecules in the cornea.

There are several limitations of this study; first, we are unable to identify the cell origin of inflammasome involvement in the cornea. Although we are able to show diminished neutrophil infiltration in NLRP3 KO mice cornea, we did not identify whether this result is the consequence of inhibition of macrophage or other corneal cell that stimulates neutrophil, or direct consequence of inflammasome inhibition in neutrophil. While regulation of inflammasome and subsequent IL-1β processing has been extensively studied in macrophages^1^, the molecular mechanisms leading to IL-1β maturation via inflammasome activation had not been addressed in neutrophils for a long time. However, recent studies showed NLRP3-derived IL-1β processing in neutrophil in inflammation status^30^. Particularly, recent report indicated that neutrophil is the predominant cell in murine model of keratitis regarding NLRP3/ASC inflammasome and caspase-1 activation, and subsequent IL-1β processing^31^. Moreover, protein depletion of not only macrophage/monocyte but also of neutrophil reveals that both cell types are widely involved in inflammasome-derived inflammation mechanisms^32–34^. And second, although we are able to show the up-regulation of caspase-1, indispensable component of NLRP3 inflammasome, in both alkali burn injury model and LPS application model, we are not able to show the cell origin of caspase-1. Although several reports in the cornea demonstrated the broad caspase-1 expression in neutrophils, macrophages, and corneal resident cells, neutrophils seem to be the predominant cell of caspase-1 expression in infectious keratitis model using NLRP3 KO mice^31,35^. Further study should be needed to elucidate other cell types involvement.

In conclusion, using alkali burn injury and LPS treatment as models of DAMP- and PAMP-induced sterile corneal inflammation, respectively, we found a pro-inflammatory role of the NLRP3 inflammasome in corneal inflammation, as evidenced by NLRP3-dependent development of corneal opacity, neutrophil infiltration, and up-regulation of IL-1β and MMP-9 expression.

## Methods

Animal experiments were performed following the recommendations of the ARVO Statement for the Use of Animals in Ophthalmic and Vision Research and were approved by the Animal Care and Use Committee at Nihon University School of Medicine

### Mouse models of corneal inflammation

Fifty eyes of 50 female C57BL/6J and 30 eyes of 30 female Nlrp3^*−/−*^ mice were examined in the experiments. The mice were anesthetized with intraperitoneal injection of mixture of medetomidine (0.3 mg/kg), midazolam (4 mg/kg), and butorphanol (5 mg/kg). Oxybuprocaine 0.4% eyedrop (Benoxil®, Santen Pharmaceutical, Osaka, Japan) was used for local anesthesia.

LPS application model was made by modifying the previous report; briefly, corneal epithelial abrasions approximately 1-mm diameter were made with a blade, and we added 40 μg of LPS in 2 μL. Thereafter, an antibiotic ointment (Tarivid®; Santen Pharmaceutical) was instilled^9^.

Corneal alkali burns were made by exposing the cornea to a filter paper of 1.5-mm diameter for 30 s^36^. The filter paper had been pre-soaked in 1 mol/l-sodium hydroxide solution. After the filter paper was removed from the cornea, the eye was rinsed with sterilized saline and an antibiotic ointment was instilled.

### Measurement of corneal opacity

The frontal view of alkali burned corneas was photographed with a digital camera (Coolpix 4500, Nikon, Tokyo, Japan) on day 7 after injury. The corneal opacity after alkali burn injury was assessed by modifying the previous report as following; 0: clear cornea graft, 1: minimal, superficial (not involving stroma) opacity, pupil margin and iris texture is readily visible, 2: minimal to moderate opacity including corneal stroma, pupil margin and iris texture is visible, 3: moderate stromal opacity, only pupil margin visible, 4: intense stromal opacity, only outline of pupil is visible, 5: severe stromal opacity, anterior chamber is invisible^37^.

### Immunohistochemistry

The mice were euthanatized by cervical dislocation under general anesthesia with intraperitoneal injection of mixture of medetomidine (0.3 mg/kg), midazolam (4 mg/kg), and butorphanol (5 mg/kg) on day 2 after LPS application, and on day 7 after alkali burn injury. Enucleated eyes were embedded (Tissue-Tek OCT Compound; Sakura Finetek Japan, Tokyo, Japan), and a 7-μm-thick section was made using a cryostat (HM505; Microm, Walldorf, Germany). These thin sections were stained by anti-Gr-1 antibody (marker of neutrophils, dilution 1:100, BD Biosciences, Franklin Lakes, NJ), anti-IL-1β antibody (dilution 1:100, Abcam, Cambridge, United Kingdom), and anti-MMP-9 antibody (dilution 1:250, Abcam) as a first antibody overnight in 4 °C, respectively. They were then stained by the indirect enzyme-antibody method (avidinebiotin complex method; Histofine®, Nichirei Biosciences, Tokyo, Japan). The section, obtained from enucleated cornea, was examined by light microscopy (BH-2, Olympus, Tokyo, Japan). Each section was observed at a working magnification of X 200 (X 20 objective lens and X 10 ocular lens; 0.723 mm2 field of view). Central cornea stroma in each mouse was observed and the cells with positive brown staining in the corneal stroma were counted.

### Laser capture microdissection and PCR analysis

The sections using embedded eye described above were cut at a thickness of 2 μm with a cryostat onto membrane-based laser microdissection slides (Leica Microsystems, Wetzlar, Germany). Corneal stroma was collected from the sections using an AS LMD laser microdissection system (Leica), and total RNA was prepared from the collected specimens using an RNeasy kit (Qiagen, Valencia, CA). RNA was subjected to reverse transcription (RT)-PCR analysis with a One-Step RT-PCR kit based on the Platinum Taq system (Invitrogen, Carlsbad, CA).

Because corneal alkali burn evokes massive inflammatory reaction, quantitative RT-PCR array analysis was performed to profile and determine the expression pattern of various genes. To determine and compare the affected genes related to inflammasome in non-treated cornea and alkali burned cornea, we employed RT² Profiler™ PCR Array Mouse Inflammasomes (PAMM-097ZC). For the comparative study of gene profiling between WT mice and NLRP3 KO mice with corneal alkali burn, we used the RT² Profiler™ PCR Array Mouse Inflammatory Response & Autoimmunity (PAMM-077). Each array contains 84 target genes and 5 housekeeping genes. The housekeeping genes (Actb, B2m, Gapdh, Gusb and Hsp90ab1) were used to normalize the data and to generate the ΔCt values; the ΔΔCt method was used to compare and calculate fold changes between non-treated and alkali burned mice, or WT and NLRP3 KO mice. The up-regulated or down-regulated genes more than 2 folds in all studied samples were extracted.

Furthermore, to determine essential gene expression, quantitative PCR was performed (TaqMan Universal PCR Master Mix and FAM-MGB dye-labeled predesigned primers [Applied Biosystems, Foster City, CA]) for NLRP3 (assay ID Mm00840904_m1), caspase-1 (assay ID Mm00438023_m1), PYCARD (assay ID Mm00445747_g1), IL-1β (assay ID Mm00434228_m1), and MMP-9 (assay ID Mm00442991_m1). PCR conditions were 2 minutes at 50 °C and 10 minutes at 95 °C, followed by 35 cycles at 95 °C for 15 seconds and 60 °C for 1 minute (ABI PRISM 7900 HT; Applied Biosystems). Results were analyzed by the comparative threshold cycle (CT) method. The relative expression level of each sample was expressed as fold change from normal control.

### Statistics

In alkali burn model, the opacity grade was compared between WT mice and NLRP3 KO mice using the Mann Whitney U-test. In immunohistochemistry of 2 types of sterile inflammation model, the positive cell counts were analyzed by the Mann Whitney U-test and a P value of <0.05 was considered statistically significant.

## Data Availability

The datasets generated during and/or analyzed during the current study are available from the corresponding author on reasonable request.
